# Trends in the Use of Neuraxial vs. General Anaesthesia for Transurethral Resection of the Prostate

**DOI:** 10.4274/TJAR.2026.262690

**Published:** 2026-10-30

**Authors:** Roman Gorchs, Vaidya Parthasarathy, Evan Burchfiel, Nitin Prabakaran, Ali Khalifa

**Affiliations:** 1 Baylor College of Medicine School of Medicine, Department of Student Affairs, Houston, TX, USA; 2 Baylor College of Medicine, Department of Anaesthesiology, Houston, TX, USA

**Keywords:** General anaesthesia, spinal block, transurethral resection of prostate, prostatic hyperplasia, aged

## Abstract

**Objective:**

Historically, neuraxial anaesthesia (NA) was preferred for transurethral resection of the prostate (TURP) to allow monitoring for TURP syndrome, but modern surgical techniques have largely eliminated this risk. We aimed to characterise trends in anaesthesia utilisation for TURP over a 15-year period and to identify patient factors associated with anaesthetic technique selection.

**Methods:**

We performed a retrospective cohort study of patients undergoing primary TURP using the ACS-NSQIP database (2009-2023). Multivariable logistic regression identified predictors of the use of general anaesthesia (GA) versus NA. Subgroup analyses examined trends by patient health status and age.

**Results:**

Of 73,214 patients identified, GA utilisation increased significantly from 69.9% in 2009 to 88.8% in 2023 (*P* < 0.001). In multivariable analysis, each successive year was associated with a 15% increase in the odds of receiving GA [odds ratio (OR) 1.15, 95% confidence interval (CI) 1.14-1.15]. Black patients (OR 1.65, 95% CI 1.47-1.87) had higher odds of GA than White patients, and Hispanic patients (OR 1.51, 95% CI 1.38-1.64) had higher odds of GA than non-Hispanic patients. Conversely, older age, chronic obstructive pulmonary disease (OR 0.77, 95% CI 0.71-0.84), and diabetes (OR 0.88, 95% CI 0.84-0.93) were associated with decreased GA use. The shift towards GA was significantly more pronounced among medically complex patients (American Society of Anesthesiologists 3-4) than among less medically complex patients (*P*=0.005).

**Conclusion:**

The anaesthetic management of TURP has evolved over the last 15 years, and GA has further solidified its status as the dominant modality. This evolution likely reflects the diminished risk of TURP syndrome, allowing practice to be driven by airway control and efficiency rather than by historical safety constraints.

**Main Points**

• The gap in usage between general and neuraxial anaesthesia has widened significantly.

• General anaesthesia has solidified its status as the primary choice for transurethral resection of the prostate.

• Neuraxial anaesthesia has become an infrequent choice for transurethral resection of the prostate.

• Chronic obstructive pulmonary disease and diabetes remain key drivers in the choice of neuraxial anaesthesia.

## Introduction

Benign prostatic hyperplasia (BPH) is one of the most common clinical diagnoses in ageing men, with a prevalence that increases dramatically with age, affecting approximately 50% of men over 50 and over 80% of men over 70.^1^ The clinical significance of BPH stems from its role as the leading cause of lower urinary tract symptoms (LUTS), which can profoundly impair quality of life.^1^ For decades, transurethral resection of the prostate (TURP) has been the surgical gold standard for treating moderate-to-severe LUTS secondary to BPH, and it remains the benchmark against which newer invasive treatments are measured.^2, 3, 4, 5^

The choice of anaesthesia for TURP, almost exclusively general anaesthesia (GA) or neuraxial anaesthesia (NA), has historically been influenced by the risks inherent in conventional monopolar TURP (M-TURP). M-TURP necessitates the use of a hypotonic irrigation fluid, which, upon absorption, can lead to TURP syndrome. This rare but potentially fatal complication is characterised by hypervolemia and dilutional hypo-osmolality.^6^ The early neurological symptoms of TURP syndrome are masked by GA, which led to a long-standing clinical preference for spinal anaesthesia to allow for intraoperative monitoring of the patient’s mental status.^7, 8^

However, the widespread adoption of modern bipolar and laser-based surgical techniques has fundamentally altered this risk-benefit calculation. By enabling the use of isotonic saline as the irrigation fluid, these newer technologies have virtually eliminated the risk of the severe dilutional hypo-osmolality that defines TURP syndrome.^6^ This technological evolution has profound implications for anaesthetic practice, as the primary safety imperative that once heavily favoured NA has been significantly diminished. With this historical constraint lifted, factors such as guaranteed patient immobility and absolute control over ventilation may now weigh more heavily in the decision-making process, potentially favouring the use of GA.

The anaesthetic management of TURP has been explored in the NSQIP database, with prior studies comparing clinical outcomes between different anaesthetic techniques.^9, 10^ Some of this work has also reported the aggregate utilisation rates for each anaesthetic modality across multi-year study windows.^10, 11^ Other research has presented anaesthesia data as a secondary characteristic in analyses of surgical trends, but without a dedicated analysis of how anaesthetic practice itself has evolved.^11^ However, no published study has conducted a dedicated longitudinal analysis of year-over-year trends in anaesthesia utilisation for TURP. This distinction is important because an aggregate rate fails to show the direction of change in practice patterns over time. As a result, a contemporary analysis of how national practice patterns have evolved remains a critical gap in knowledge. This study aims to fill that gap by describing these trends over the 15-year period from 2009 to 2023.

## Methods

### Study Design and Data Source

We performed a retrospective cohort study using the American College of Surgeons National Surgical Quality Improvement Program (ACS-NSQIP) database. The NSQIP is a large, multicenter, outcomes-based registry that collects data on preoperative risk factors, intraoperative variables, and 30-day postoperative outcomes from hospitals participating in the ACS-NSQIP.

### Ethics Committee

This study was approved by the Institutional Review Board at Baylor College of Medicine (approval no: H-58079, date: 14.10.2025). The requirement for informed consent was waived due to the retrospective nature of the study and the use of de-identified data.

### Study Population

We queried the NSQIP database to identify all adult patients (≥18 years of age) who underwent a primary TURP, identified by current procedural terminology code 52601, between January 1, 2009, and December 31, 2023. We excluded patients with an American Society of Anesthesiologists (ASA) physical status classification of 5, those classified as emergent or with preoperative sepsis. To create a cohort focused on benign disease, patients with a postoperative diagnosis of prostate cancer [identified using International Classification of Diseases (ICD)-9 code 185 and ICD-10 code C61] or with disseminated cancer flagged in the NSQIP registry were excluded (Figure 1).

### Study Variables

The primary variable of interest was the type of anaesthesia administered, as recorded in the ANESTHES variable. We categorised this variable into two groups for comparison: GA and NA, with the NA group encompassing cases recorded as “spinal”, “epidural”, or “regional”. To create a clear comparison between these two primary techniques, the small number of cases performed under monitored anaesthesia care (MAC) or local anaesthesia were excluded from the final analysis.

Additional patient and procedural variables were collected to describe the study cohorts and to serve as covariates in multivariable models. These included patient demographics [age, race, Hispanic ethnicity, body mass index (BMI) category], preoperative comorbidities [ASA class, diabetes mellitus, hypertension requiring medication, congestive heart failure, chronic obstructive pulmonary disease (COPD), smoking status, dialysis dependence], and the year of surgery. All variables were used to perform a descriptive comparison between the two anaesthetic groups (Table 1). The multivariable model described in the statistical analysis section (Table 2) retained year of surgery, age, race, Hispanic ethnicity, ASA class, diabetes mellitus, hypertension, congestive heart failure, COPD, dialysis dependence, and BMI category. These covariates were pre-specified based on documented or biologically plausible relevance to anaesthetic technique selection.

### Statistical Analysis

All statistical analyses were performed using R statistical software (version 4.3.1, R Foundation for Statistical Computing). A two-sided *P* value of < 0.05 was considered statistically significant.

First, a longitudinal trend analysis was performed to assess the primary objective. The proportion of TURPs performed under GA versus NA was calculated for each calendar year from 2009 to 2023, and a line graph was generated to visualise this trend (Figure 2). The year of surgery was modelled as a continuous variable throughout the analysis. The statistical significance of the temporal trend reported in the trends in anaesthesia utilisation section was assessed using the coefficient for year of surgery from an unadjusted logistic regression model with anaesthesia type (GA vs. NA) as the outcome; this unadjusted association was then re-examined in a model adjusted for the covariates listed above, with the adjusted estimate reported separately in the predictors of anaesthesia choice section (Table 2).

Second, a descriptive analysis was performed to compare the baseline characteristics of the two anaesthetic groups. Categorical variables were compared using the chi-square test; continuous variables were compared using the Student’s t-test. To assess clinically meaningful imbalances between groups, the standardised mean difference was calculated for each variable.

Third, to identify factors independently associated with the choice of anaesthesia, a multivariable logistic regression model was constructed. The outcome was receipt of GA (vs. NA), and the model included year of surgery, patient demographics, and key comorbidities as predictors.

Finally, to explore whether the trend in anaesthesia utilisation differed across patient populations, a subgroup analysis was performed. The cohort was stratified by both patient health status (ASA class 1-2 vs. 3-4) and age (<70 vs. ≥70 years). The longitudinal trend analysis was then repeated for each subgroup. To formally test for a difference in trends between the groups, an interaction term between the year of surgery and the subgroup variable was added to the multivariable logistic regression model. All multivariable models were constructed using a complete-case analysis; the extent of missing data and the number of patients excluded on this basis are reported in the patient characteristics section.

## Results

### Patient Characteristics

After applying the inclusion and exclusion criteria, a final cohort of 73,214 patients who underwent TURP between 2009 and 2023 was identified. Of these, 59,193 (80.8%) received GA and 14,021 (19.2%) received NA. The study flow diagram (Figure 1) shows the number of patients excluded at each step: 13 for ASA class 5; 1,264 for emergent cases; 318 for preoperative sepsis; 1,575 for disseminated cancer; 5,321 for prostate cancer (ICD-9 185/ICD-10 C61); 2,665 for MAC or local anaesthesia; and 214 for missing/uninterpretable ASA class, yielding the final analytic cohort of 73,214 patients. Among the covariates included in the multivariable model, only age had missing values (1,324 patients; 1.81%); all other covariates were complete. A total of 1,324 patients were excluded on a complete-case basis, leaving 71,890 patients in the final multivariable regression model (Table 2).

The baseline characteristics of the two groups differed significantly (Table 1). Patients who received NA on average were older than those who received GA (mean ages 72.55 and 70.32 years, *P* < 0.001). The NA cohort also had a higher burden of comorbidities, notably a greater proportion of ASA class 4 patients (6.0% vs. 4.3%, *P* < 0.001) and a higher prevalence of COPD (7.1% vs. 5.4%, *P* < 0.001). Conversely, congestive heart failure was more prevalent in the GA cohort (2.2% vs. 1.6%, *P* < 0.001).

### Trends in Anaesthesia Utilisation

Over the 15-year study period, a significant and sustained trend toward increased use of GA and a corresponding decrease in use of NA for TURP procedures was observed (Figure 2). The proportion of TURPs performed under GA increased from 69.9% in 2009 to 88.8% in 2023. Conversely, the proportion of procedures performed under NA decreased from 30.1% in 2009 to 11.2% in 2023. This trend was statistically significant over time (*P* < 0.001).

### Predictors of Anaesthesia Choice

The multivariable logistic regression analysis identified several independent predictors of receiving GA over NA (Table 2). After controlling for other factors, each successive year of surgery was associated with a 15% increase in the odds of receiving GA (OR 1.15, 95% CI 1.14-1.15). Older age was associated with decreased odds of receiving GA (OR 0.98 per year, 95% CI 0.97-0.98).

Compared with White patients, Black patients had significantly higher odds of receiving GA (OR 1.65, 95% CI 1.47-1.87), as did Hispanic patients compared with non-Hispanic patients (OR 1.51, 95% CI 1.38-1.64). ASA class was non-linearly associated with anaesthetic choice. Compared with ASA class 1 patients, patients classified as ASA class 2 and ASA class 3 had higher odds of receiving GA (OR 1.18 and 1.21, respectively), whereas the odds for ASA class 4 patients did not differ significantly from those for ASA class 1 patients (OR 0.97, 95% CI 0.83-1.12). Conversely, patients with a history of COPD (OR 0.77, 95% CI 0.71-0.84) or diabetes (OR 0.88, 95% CI 0.84-0.93) had significantly lower odds of receiving GA.

### Subgroup Analysis of Anaesthesia Trends

A subgroup analysis was performed to determine whether the trend in anaesthesia utilisation differed by patient health status or age. The trend towards increased GA use differed significantly between patients with lower ASA scores (ASA 1-2) and those with higher ASA scores (ASA 3-4) (Figure 3). Although ASA class 4 patients were proportionally more common in the NA group (Table 1), the trend of increasing GA utilisation over the 15-year period was significantly more pronounced in this group than in less medically complex patients (*P* value for interaction =0.005). In contrast, the trend was consistent across both younger (<70 years) and older (≥70 years) patient populations (Figure 4), with no statistically significant difference observed between the two groups (*P* value for interaction =0.991). Both subgroup analyses (Figures 3 and 4) were restricted to the 71,890 patients with non-missing age data. The primary trend analysis (Figure 2) and Table 1 remain unaffected because they do not depend on this exclusion.

## Discussion

In this large, multi-institutional study of over 73,000 patients, we found a profound and sustained shift in anaesthetic practice for TURP between 2009 and 2023. The data clearly show that GA has become the dominant modality, increasing from just over two-thirds of cases in 2009 to nearly 90% in the modern era. This trend toward GA remained highly significant even after adjusting for a wide range of patient demographics and comorbidities.

While several factors may contribute to this trend, one plausible and clinically coherent contributor is the procedural innovation, which has transformed TURP over time. The NSQIP database does not capture the specific surgical technology used in each case; therefore, this interpretation should be regarded as a likely explanation rather than a finding directly supported by our data. Historically, the risk of the rare but potentially fatal TURP syndrome made NA the preferred technique, as it allowed for the intraoperative monitoring of a patient’s mental status to detect early signs of hypo-osmolality.^7, 8^ However, the widespread adoption of bipolar and laser-based technologies, which permit the use of isotonic saline for irrigation, has virtually eliminated this risk. The neutralisation of the primary safety concern that once favoured NA has liberated the choice of anaesthetic, allowing other factors, such as procedural efficiency, guaranteed patient immobility, and absolute airway control, to potentially favour the use of GA.

Interestingly, our analysis reveals that while a national trend towards GA exists, nuanced clinical judgement remains a critical component of anaesthetic choice. Compared with those receiving GA, patients selected for NA were, on average, slightly older and more likely to have comorbidities, such as COPD and diabetes mellitus. Prior studies indicate that spinal anaesthesia may decrease morbidity in patients with COPD, which may underlie anaesthesiologists’ preference for NA in this population.^12^ Additionally, another study that utilised the NSQIP database found that NA offered a statistically significant advantage in 30-day outcomes.^9^ These findings demonstrate that clinicians may be selectively using NA for a population they perceive to be at higher risk.

Our subgroup analysis reveals that the shift towards GA is not uniform. The trend towards GA was significantly more pronounced in medically complex patients (ASA 3-4), which represents an evolution in the anaesthetic management of this higher-risk cohort. In contrast, the trend towards GA has progressed similarly across age groups, with no statistically significant difference in the rate of change between younger and older patients. These findings suggest that the factors influencing this national trend are more closely related to patient health status than to age.

Prior research using the NSQIP database has analysed anaesthesia trends in other surgical specialties, providing valuable context for our findings. In oncologic breast surgery, a 15-year analysis found a significant trend towards increased GA use for lumpectomy procedures, mirroring the shift we observed in TURP.^13^ However, this pattern is not consistent with orthopaedic procedures. For hip fracture repair, the opposite trend was observed between 2007 and 2017, with a notable increase in the use of spinal anaesthesia.^14^ Moreover, for total hip and knee arthroplasty, the utilisation of spinal anaesthesia was found to be largely unchanged between 2011 and 2018, despite being considered a grossly underutilised gold standard.^15^ These contrasting trajectories suggest that procedural evolution, patient characteristics, and established clinical pathways unique to each field are the primary drivers of anaesthetic selection.

### Study Limitations

This study has multiple limitations inherent in its retrospective design, which used a large administrative database. The NSQIP database does not contain information on the specific surgical technology used (e.g., monopolar, bipolar, laser); therefore, we cannot directly attribute the observed trend to the adoption of newer technologies. Additionally, we lack data on surgeon or patient preferences, operating room scheduling pressures, or the specific reasons behind each anaesthetic choice. As with any observational analysis, residual confounding from unmeasured factors such as institutional practice patterns or individual anaesthetist preference cannot be excluded and may partly account for the associations observed. The number of hospitals contributing to the NSQIP database grew substantially over the study period. Therefore, the cohort from the initial study period may be less representative of national practice patterns compared to the cohort from more recent years.^16^

## Conclusion

In conclusion, the anaesthetic landscape for TURP has been fundamentally reshaped over the last 15 years. The practice has moved away from a choice dictated by the risk of a single, now-rare complication and toward a choice influenced by a modern balance among patient factors, procedural demands, and demographic patterns. This study documents a paradigm shift in anaesthetic practice for TURP that mirrors the broader technological transformation of the procedure.

## Ethics

**Ethics Committee Approval: **This study was approved by the Institutional Review Board at Baylor College of Medicine (approval no: H-58079, date: 14.10.2025).

**Informed Consent: **The requirement for informed consent was waived due to the retrospective nature of the study and the use of de-identified data.

## Figures and Tables

**Figure 1:**
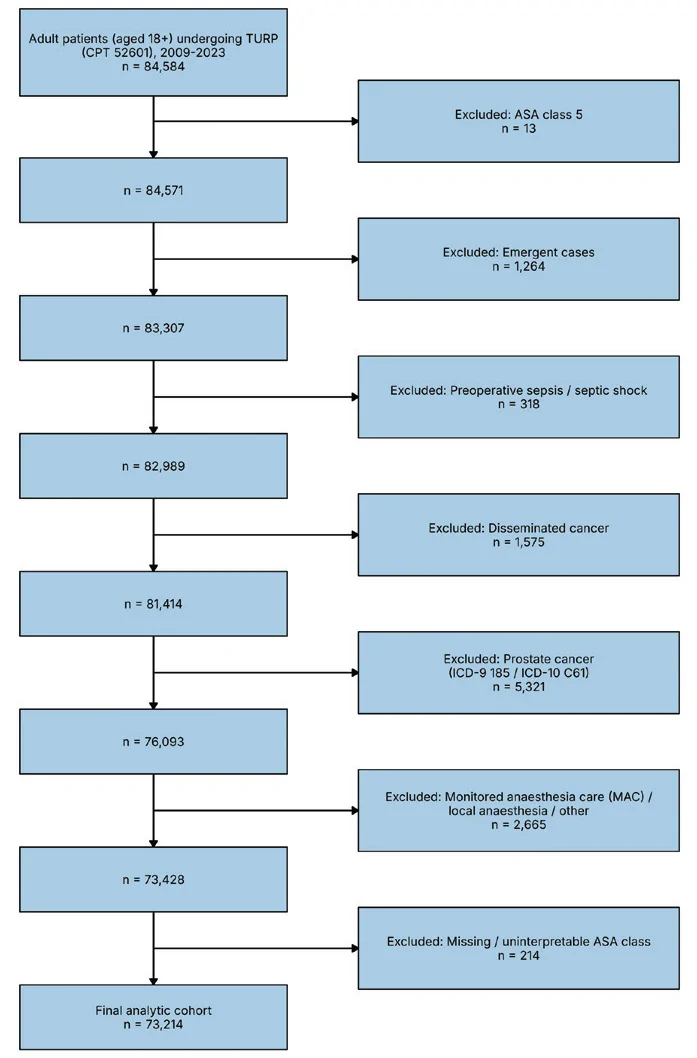
Study cohort selection flow diagram. Flow diagram illustrating the sequential application of inclusion and exclusion criteria used to derive the final study cohort from the ACS-NSQIP database (2009-2023). ACS-NSQIP, American College of Surgeons National Surgical Quality Improvement Program; ASA, American Society of Anesthesiologists; TURP, transurethral resection of the prostate; CPT, current procedural terminology; ICD, International Classification of Diseases.

**Figure 2:**
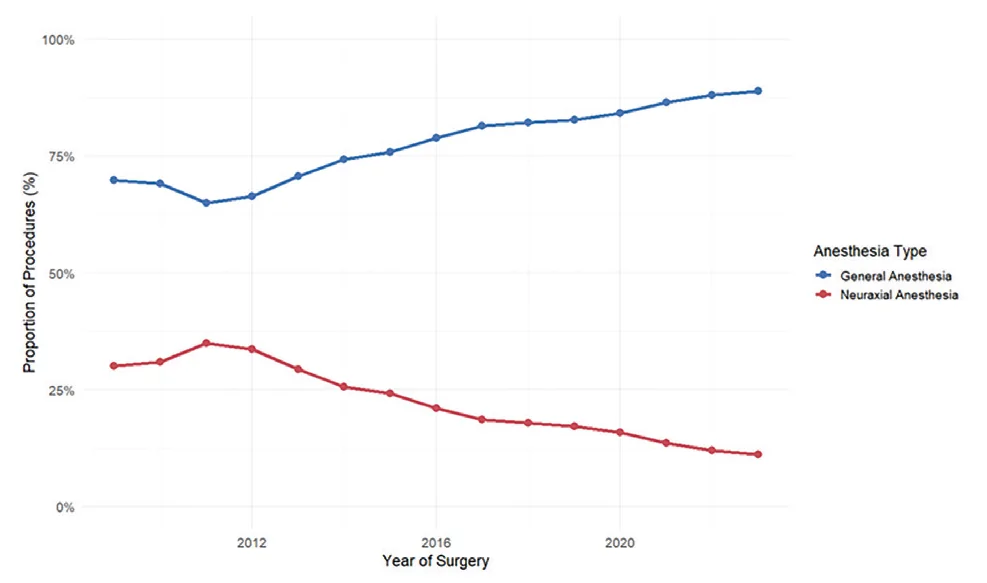
Trends in type of anaesthesia for transurethral resection of the prostate (TURP) (2009-2023). The line graph illustrates the annual proportion of TURP procedures performed under general anaesthesia (blue line) versus neuraxial anaesthesia (red line) within the ACS-NSQIP database. Over the 15-year study period of 2009-2023, there was a significant increase in the use of general anaesthesia with a concurrent decline in neuraxial anaesthesia. ACS-NSQIP, American College of Surgeons National Surgical Quality Improvement Program.

**Figure 3:**
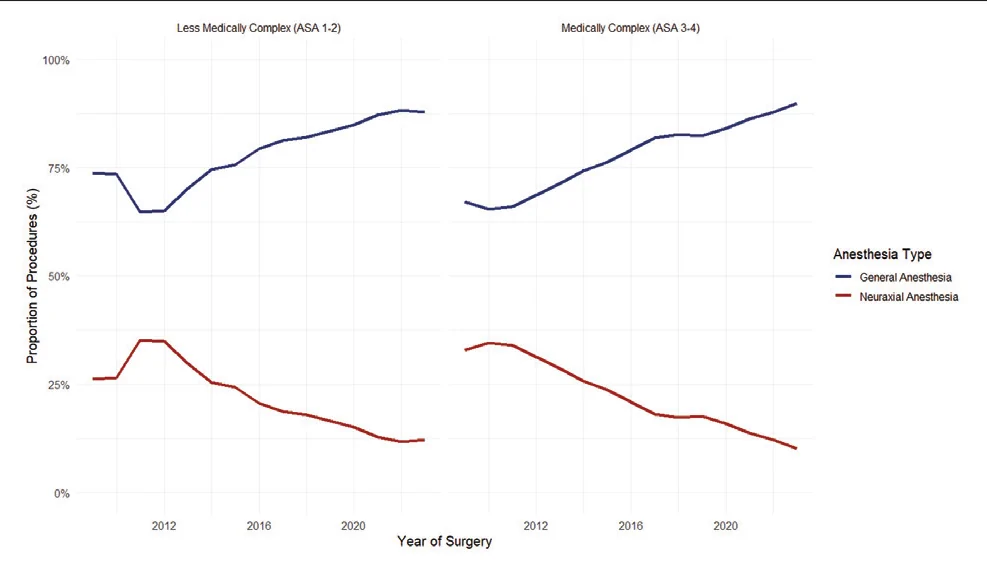
Trends in type of anaesthesia by patient health status. The figure displays the longitudinal trends in anaesthesia choice for healthier patients (ASA class 1-2, left panel) and medically complex patients (ASA class 3-4, right panel). The blue line represents general anaesthesia, and the red line represents neuraxial anaesthesia. ASA, American Society of Anesthesiologists.

**Figure 4:**
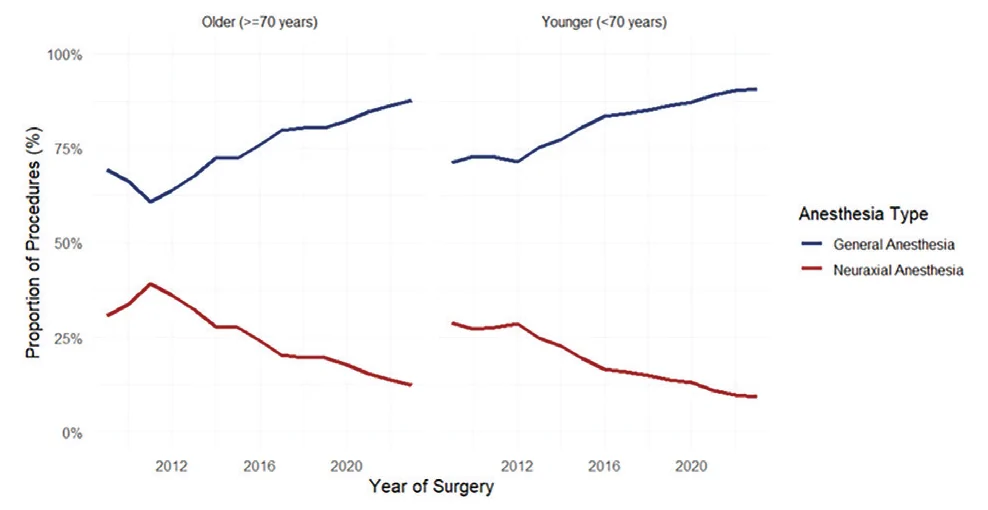
Trends in type of anaesthesia by patient age group. The figure displays longitudinal trends in anaesthesia choice for older patients (≥70 years, left panel) and younger patients (<70 years, right panel). The blue line indicates general anaesthesia, and the red line indicates neuraxial anaesthesia.

**Table 1. Demographics for Patients Receiving General Anaesthesia versus Neuraxial During Their Procedure (2009-2023):** 

**n**	**General anaesthesia**	**Neuraxial anaesthesia**	***P* value**	**SMD**
	**59193**	**14021**	-	-
Age [mean (SD)]	70.32 (9.08)	72.55 (8.58)	<0.001	0.252
**BMI** **category** **(%)**			<0.001	0.146
Normal	14481 (24.5)	3964 (28.3)	-	-
Obese	19267 (32.5)	3928 (28.0)	-	-
Overweight	24289 (41.0)	5639 (40.2)	-	-
Underweight	1156 ( 2.0)	490 (3.5)	-	-
**Race** **(%)**			<0.001	0.802
Asian	2080 (3.5)	478 (3.4)	-	-
Black	4648 (7.9)	323 (2.3)	-	-
Other/unknown	12648 (21.4)	7982 (56.9)	-	-
White	39817 (67.3)	5238 (37.4)	-	-
Hispanic	5308 (9.0)	703 (5.0)	<0.001	0.156
**ASA** **class** **(%)**			<0.001	0.126
1-No disturb	1323 (2.2)	542 ( 3.9)	-	-
2-Mild disturb	25399 (42.9)	5902 (42.1)	-	-
3-Severe disturb	29937 (50.6)	6738 (48.1)	-	-
4-Life threat	2534 (4.3)	839 (6.0)	-	-
Diabetes mellitus (%)	13194 (22.3)	3259 (23.2)	0.015	0.023
Hypertension requiring medication (%)	35736 (60.4)	8272 (59.0)	0.003	0.028
Congestive heart failure (%)	1281 (2.2)	221 (1.6)	<0.001	0.043
COPD (%)	3219 (5.4)	1002 (7.1)	<0.001	0.070
Smoking history (%)	6259 (10.6)	1441 (10.3)	0.311	0.010
Dialysis (%)	319 (0.5)	66 (0.5)	0.348	0.010

SD, standard deviation; BMI, body mass index; ASA, American Society of Anesthesiologists; COPD, chronic obstructive pulmonary disease; SMD, standardised mean difference

**Table 2. Multivariable Analysis of Factors Predicting Receipt of General Anaesthesia over Neuraxial Anaesthesia for TURP:** 

-	**Odds ratio**	***P* value**	**95% CI lower**	**95% CI upper**
Year of operation	1.15	<0.001	1.14	1.15
Age	0.98	<0.001	0.97	0.98
Race-Asian	0.52	<0.001	0.47	0.58
Race-Black	1.65	<0.001	1.47	1.87
Race-other/unknown	0.19	<0.001	0.18	0.19
Hispanic	1.51	<0.001	1.38	1.64
ASA class 2	1.18	0.004	1.05	1.33
ASA class 3	1.21	0.002	1.07	1.36
ASA class 4	0.97	0.665	0.83	1.12
Diabetes mellitus	0.88	<0.001	0.84	0.93
Hypertension requiring medication	1.01	0.659	0.97	1.06
Congestive heart failure	1.18	0.045	1.01	1.40
COPD	0.77	<0.001	0.71	0.84
Dialysis	1.12	0.445	0.84	1.53
BMI category-obese	1.11	<0.001	1.05	1.18
BMI category-overweight	1.08	0.003	1.03	1.14
BMI category-underweight	0.87	0.032	0.77	0.99

Reference categories: White race, non-Hispanic ethnicity, ASA class 1, normal BMI, and absence of each comorbidity. Year of operation and age were modelled as continuous variables (OR per one-year increase). Model based on complete-case analysis (n = 71,890); 1,324 patients with missing age were excluded

BMI, body mass index; CI, confidence interval; COPD, chronic obstructive pulmonary disease; ASA, American Society of Anesthesiologists; TURP, transurethral resection of the prostate; OR, odds ratio
